# Correlation between Optic Nerve Parameters Obtained Using 3D Nonmydriatic Retinal Camera and Optical Coherence Tomography: Interobserver Agreement on the Disc Damage Likelihood Scale

**DOI:** 10.1155/2014/931738

**Published:** 2014-04-02

**Authors:** Jae Wook Han, Soon Young Cho, Kui Dong Kang

**Affiliations:** Department of Ophthalmology, Incheon St. Mary's Hospital, College of Medicine, The Catholic University of Korea, Dongsoo-Ro 58, Bupyung-Gu, Incheon 403-720, Republic of Korea

## Abstract

*Purpose*. To compare stereometric parameters obtained by three-dimensional (3D) optic disc photography and optical coherence tomography (OCT) and assess interobserver agreement on the disc damage likelihood scale (DDLS). *Methods*. This retrospective study included 190 eyes from 190 patients classified as normal, glaucoma suspect, or glaucomatous. Residents at different levels of training completed the DDLS for each patient before and after attending a training module. 3D optic disc photography and OCT were performed on each eye, and correlations between the DDLS and various parameters obtained by each device were calculated. *Results*. We found moderate agreement (weighted kappa value, 0.59 ± 0.03) between DDLS scores obtained by 3D optic disc photography and the glaucoma specialist. The weighted kappa values for agreement and interobserver concordance increased among residents after the training module. Interobserver concordance was the poorest at DDLS stages 5 and 6. The DDLS scored by the glaucoma specialist had the highest predictability value (0.941). *Conclusions*. The DDLS obtained by 3D optic disc photography is a useful diagnostic tool for glaucoma. A supervised teaching program increased trainee interobserver agreement on the DDLS. DDLS stages 5 and 6 showed the poorest interobserver agreement, suggesting that caution is required when recording these stages.

## 1. Introduction

Glaucoma is group of optic neuropathies associated with characteristic structural damage to the optic nerve as a result of various pathological processes that lead to visual dysfunction [1]. Glaucoma is a debilitating disease that causes blindness if left untreated; thus, early diagnosis is crucial. Although early detection and treatment of glaucoma may halt progression of the disease, patients with early glaucoma often do not seek medical help because they do not notice changes in their vision. Thus, recent studies have focused on the early detection and treatment of this debilitating disease.

In general, diagnostic tests for glaucoma include the measurement of intraocular pressure (IOP), the visual field test, and examination of the optic nerve head. Although elevated IOP is one of the most important risk factors for glaucoma, the damage threshold varies among individuals and diurnal fluctuations can conceal IOP spikes. The visual field test is limited as an early diagnostic tool because defects in the visual field occur only following significant ganglion cell loss [2–4]. Furthermore, examination of the optic nerve head has disadvantages such as high interobserver variability and low reproducibility. Several new methods for the evaluation of the optic nerve head have been proposed to overcome these limitations. The disc damage likelihood scale (DDLS) incorporates the size of the disc and the radial width of the neuroretinal rim into the evaluation of the optic nerve head [5]. The DDLS has been reported to provide a more accurate assessment of optic disc damage than the conventional cup/disc (C/D) ratio measurement [6], and a high correlation has been found between the DDLS and various indices obtained from Heidelberg retina tomography and optical coherence tomography (OCT) in patients with glaucoma [7, 8]. Nevertheless, interobserver variability is a potential limitation of DDLS and training on the system is essential for low interobserver variability and high reproducibility.

Recently, a three-dimensional (3D) stereographic camera (Kowa nonmyd WX 3D, Kowa, Tokyo, Japan) that provides an objective recording of the optic nerve head was developed. This device provides a software algorithm that automatically displays DDLS in its final report output. The shape of optic nerve can be visualized on a 3D display, which provides a significant advantage in terms of evaluating the depth distribution of the optic cup and neuroretinal rim. The 3D analysis option is expected to decrease interobserver variability and increase reproducibility compared with standard optic nerve head photographs.

We evaluated the validity of the DDLS option provided by this 3D stereographic camera and assessed the correlations between various stereometric optic disc parameters obtained by the 3D stereographic camera and optic disc cube parameters measured using OCT. Moreover, we provide the first report, to our knowledge, of interobserver agreement on the DDLS using kappa statistics and evidence of the usefulness of a training module that includes a detailed introduction and clinical skills for measuring DDLS.

## 2. Materials and Methods

The research was conducted according to the principles of the Declaration of Helsinki and was approved by the Institutional Review Board of Incheon St. Mary's Hospital, The Catholic University of Korea (IRB number OC13RISI0082). Our retrospective study comprised 190 eyes from 190 patients. All subjects underwent comprehensive ophthalmological examinations including medical, ocular, family history, and visual acuity testing and refraction. Standard achromatic perimetry performed with a Humphrey Field Analyzer (Zeiss Humphrey Systems, Dublin, CA, USA) using the 24-2 full-threshold test, IOP measurement, dilated slit-lamp biomicroscopy, 3D optic disc photography (Kowa nonmyd WX 3D, Kowa, Tokyo, Japan), optic nerve head, and retinal nerve fiber layer (RNFL) analysis using Cirrus OCT (Carl Zeiss Meditec Inc., Dublin, CA, USA) were performed during the same visit for each patient between March 2011 and May 2013. Following pupil dilation using tropicamide 0.5% and phenylephrine 5%, a glaucoma specialist (K.D.K) calculated the vertical and horizontal C/D ratios (uncorrected for optic disc size) using direct ophthalmoscope (Welch Allyn, Skaneateles Falls, NY, USA). The optic cup was defined on the basis of contour and the course of the small blood vessels on the disc and not on the basis of pallor. The optic disc border was defined as the inner border of the peripapillary scleral ring.

The 3D stereoscopic analysis of the optic nerve head was performed according the manufacturer's instructions. Briefly, following pupil dilation, photographic stereo pairs were captured and displayed on a 3D monitor. The optic nerve head was viewed using prism glasses, and the C/D ratio was plotted manually to determine the contour line of each structure. The 3D analysis was performed using an integrated software package. All OCT scans were acquired with a Cirrus HDOCT (version 3.0.0.64) using the Optic Disc Cube 200 × 200 protocol, which is designed to position the cube scan on the optic nerve head and is primarily used for glaucoma analysis. Only scans of good quality (signal strength better than 6, without RNFL discontinuity or misalignment, involuntary saccade, or blinking artifacts, and absence of RNFL algorithm segmentation failure without misalignment or movement artefacts) were included in the analysis.

The 190 patients were divided into three diagnostic groups: normal, glaucoma suspect, and glaucoma. The criteria for the normal group were (1) healthy subjects with no history or presence of glaucoma, no retinal pathological findings, and no intraocular surgery including laser therapy; (2) IOP ≤21 mm Hg on each visit; (3) normal Humphrey 24-2 visual field test; (4) best corrected visual acuity of 20/40 or better with refractive error between +1.00 and −3.00 diopters; (5) open angles by gonioscopy; and (6) normal-appearing optic nerve head. The criteria for glaucoma suspect eyes were (1) no history or presence of retinal pathology and no intraocular surgery including laser therapy; (2) IOP between 22 and 30 mmHg; and (3) normal visual field test. Patients who had the following findings were also categorized as glaucoma suspect, (a) asymmetric optic nerve head cupping (difference in vertical C/D ratio ≥0.2 between the eyes in the presence of a similar optic disc size) or (b) increased cupping (vertical C/D ratio >0.6). The diagnostic criteria for glaucoma were (1) an abnormal visual field defined as the presence of at least two of the following. (a) A glaucoma hemifield test outside normal limits, (b) *P* < 5% for corrected pattern standard deviation, or (c) a cluster of at least three contiguous points with *P* < 5%, including at least one of these with *P* < 1% in the pattern deviation plot. (2) One or more papillary sign as follows: (a) presence of a localized loss or thinning of the neuroretinal rim, (b) optic disk excavation, (c) vertical or horizontal C/D ratio >0.6, and (d) C/D asymmetry between the two eyes ≥0.2. Patients with a previous history of intraocular surgery, including laser therapy or undergoing a systemic therapy that could interfere with ocular hydrodynamics were excluded from the glaucoma subgroup.

Residents at different levels of training evaluated the eyes of the patients using the DDLS. As the ophthalmology residency in Korea involves 4 years training in an accredited institution, we recruited three residents from each postgraduate year (PGY) for our study (12 residents in total). The residents were asked to evaluate each patient using the DDLS with no prior training and only the DDLS index as a reference. Six residents evaluated patients on their initial visit to the clinic and the remaining six evaluated patients on their second visit (usually 3 months after the initial visit). A glaucoma specialist (K.D.K) used the DDLS to evaluate the patients on their initial visit. Following the enrollment of all subjects, a 2-h DDLS training program designed to provide detailed information about the grading system was conducted for all residents. It included a 1-h objective structured clinical examination (OSCE) followed by a 1-h lecture. The lecture consisted of an introduction to the system, instructions on its use, and a discussion of difficulties involved in scoring (such as multiplying the size of the disc by the corresponding corrective factors when using 60 or 90 diopter lenses). Following the training program, the residents were asked to reevaluate the DDLS of the participating subjects. At this point, the residents were not aware of their previous DDLS scores. Additionally, all measurements were made using a Superfield NC lens (Volk Optical, Miami, FL, USA) to avoid magnification correction errors, and “interobserver agreement” was defined as two observers exactly agreeing on the DDLS value. When analyzing the results, we incorporated the 0a and 0b stages initially proposed by Spaeth et al. [5] into one stage, 0.

The level of interobserver agreement was measured using the weighted kappa statistic because it is an appropriate chance-adjusted measure of agreement between two observers when there are more than two ordered categories of classification [9]. An analysis of variance (ANOVA) was used to evaluate the differences among the glaucoma, glaucoma suspect, and normal groups on the various parameters, and the post hoc analysis was conducted using Scheffe's test. The correlations between the DDLS value and the Cirrus OCT and 3D optic disc photography measurements were assessed using Pearson's coefficient of correlation. The statistical tests were conducted using STATA/IC (version 11.2; StataCorp LP, College Station, TX, USA), and a *P* value <0.05 was deemed to indicate statistical significance.

## 3. Results

Of the 190 eyes under study, 80 were diagnosed with glaucoma, 70 were glaucoma suspect, and 40 were without glaucoma. Patient demographics and baseline characteristics are shown in Table 1. The average 3D stereographic DDLS scores for the normal, glaucoma suspect, and glaucoma groups were 0.58 ± 0.50, 2.03 ± 0.66, and 4.23 ± 1.23, respectively. The average DDLS scores obtained by the glaucoma specialist for each group were 1.47 ± 0.56 (normal), 2.39 ± 0.78 (glaucoma suspect), and 4.91 ± 1.42 (glaucoma). Refraction error and disc diameter did not differ among the patient groups (one-way ANOVA). The mean deviation of visual field test was −2.35 ± 3.28 for the normal, −2.47 ± 2.52 for the glaucoma suspect, and −8.57 ± 8.78 for the glaucoma groups.

**Figure 1:**
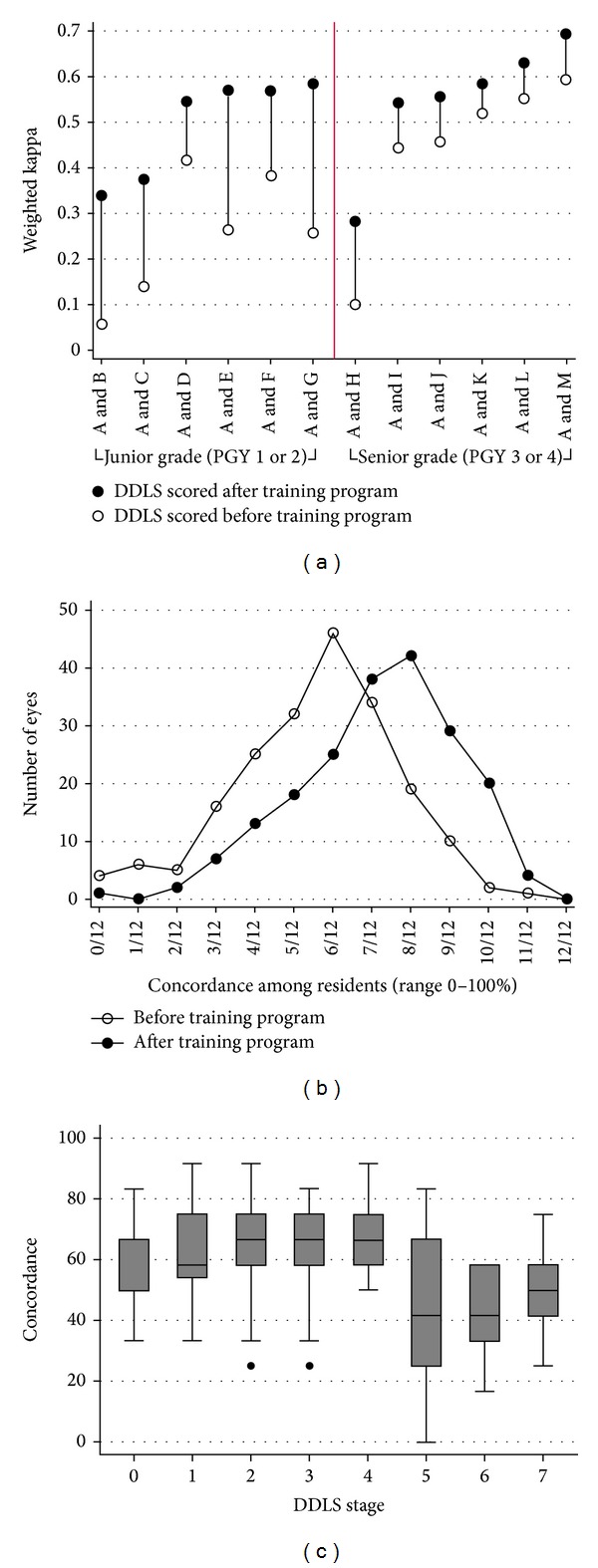
(a) The weighted kappa values for interobserver agreement on the disc damage likelihood scale (DDLS) for the 12 pairs (glaucoma specialist versus all residents). All possible pairs are plotted on the* X-*axis and the weighted kappa values are shown on the* Y-* axis. White circles indicate the weighted kappa values before the training program and black circles indicate the weighted kappa values after attending the training program. The residents were divided according to level of training: junior (PGY 1 or 2) and senior (PGY 3 or 4) as indicated by the red line. The weighted kappa values increased after the training program, and this positive effect was greater among junior residents. (b) Concordance among residents on the DDLS evaluation before (white circles) and after (black circles) the training program. Note the right shift (toward better concordance among the residents) after the training program. (c) Concordance among residents according to DDLS stage. Note that the concordance is lowest for stages 5 and 6, whereas it is good for stages 0–4.

The weighted kappa value for interobserver agreement on the DDLS between the 3D optic disc photography and glaucoma specialist assessments was 0.59 ± 0.03. The interobserver agreement between the glaucoma specialist and the residents was analyzed according to each of the 12 possible pairs (glaucoma specialist (A) versus each resident [B, C, D, E, F, G, H, I, J, K, L, and M,]; Figure 1(a)) before (white circle) and after (black circle) the DDLS training module. The weighted kappa values for interobserver agreement were higher after the training module in all pairs (Figure 1(a)). Although the variation in weighted kappa values among the pairs was substantial, the increase in interobserver agreement was higher in the junior than in the senior residents.

Figure 1(b) shows the DDLS concordance among residents before (white circles) and after (black circles) the training module. Concordance was defined as the percentage of agreement among the 12 residents on the DDLS score for each eye compared with the gold standard value (the glaucoma specialist's DDLS score). For example, 50 (6/12) percent concordance was achieved when 6 of the 12 residents were in complete agreement with the DDLS evaluation of the glaucoma specialist. Concordance values after the training program showed a right shift toward improved interobserver concordance.  Figure 1(c) shows the concordance among residents according to DDLS stage. The* X-*axis shows DDLS stages (0–7) and the* Y*-axis shows the percentage concordance among residents. The degree of concordance was the lowest on stages 5 and 6, whereas good concordance was observed on stages 0–4.

The mean values for the ophthalmoscopic examination, 3D optic disc photography, and Cirrus OCT according to diagnostic group are shown in Table 2. The average vertical C/D ratios of the normal, glaucoma suspect, and glaucoma groups were 0.48 ± 0.11, 0.57 ± 0.03, and 0.64 ± 0.11, respectively. The average C/D area ratios (obtained by 3D optic disc photography) of the normal, glaucoma suspect, and glaucoma groups were 0.25 ± 0.10, 0.33 ± 0.65, and 0.42 ± 0.12, respectively. The ANOVA revealed statistically significant differences among all parameters obtained by 3D optic disc photography with the exception of the height variation contour (*P* = 0.234). The post hoc analysis of the intervariable mean differences between the normal and glaucoma suspect groups revealed that all variables were significantly different, with the exception of the superior rim width (*P* = 0.182), inferior rim width (*P* = 0.361), and height variation contour (*P* = 0.281). Furthermore, the post hoc analysis of the intervariable mean differences between the glaucoma suspect and glaucoma groups revealed significant differences for all variables with the exception of the disc area (*P* = 0.657), cup volume (*P* = 0.285), disc volume (*P* = 0.352), mean cup depth (*P* = 0.486), maximum cup depth (*P* = 0.326), and height variation contour (*P* = 0.994).

The vertical C/D ratio, cup area, C/D area ratio, and cup volume obtained by 3D optic disc photography were positively correlated with the DDLS score of the glaucoma specialist (Pearson's correlation, Table 3). Among the parameters measured by 3D optic disc photography, the vertical C/D ratio was the most highly correlated with the DDLS (*r* = 0.623, *P* < 0.001). The superior rim width, inferior rim width, rim area, rim disc area ratio, and rim volume were negatively correlated with the DDLS (*P* < 0.001), and disc area, disc volume, mean cup depth, maximum cup depth, and height variation were not correlated with the DDLS. All of the parameters acquired by Cirrus OCT were significantly correlated with the DDLS with the exception of disc area.

**Figure 2:**
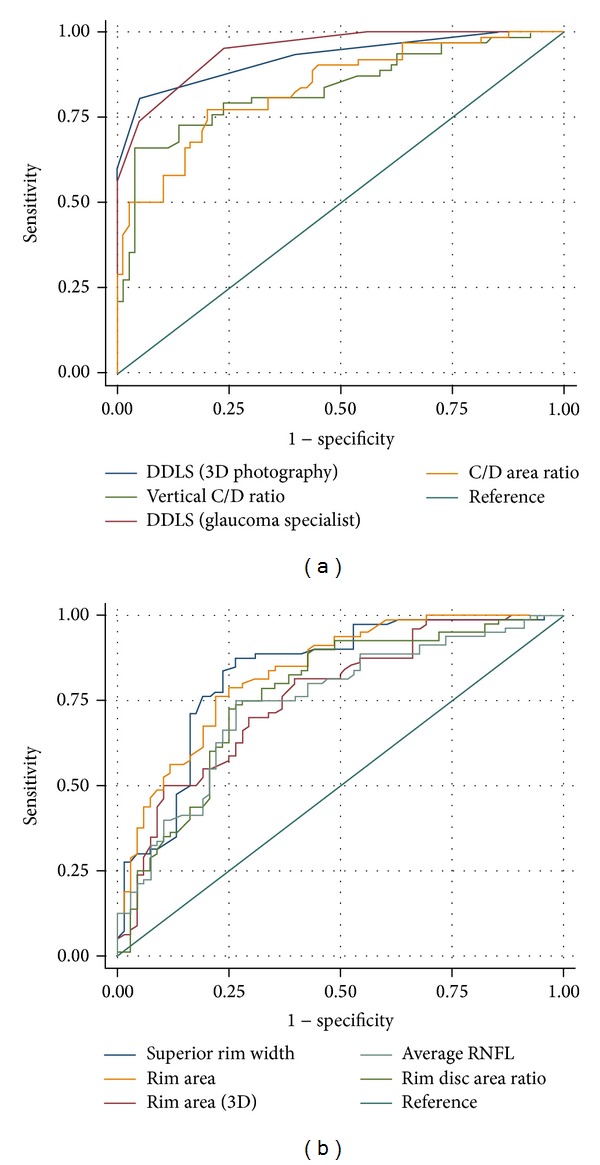
Receiver operating characteristic (ROC) curves for the discriminant functions showing the comparison of the glaucoma and normal groups. (a) The comparison of the disc damage likelihood scale (DDLS; obtained by 3D optic disc photography and the glaucoma specialist), vertical cup-to-disc (C/D) ratio, C/D area ratio, and superior rim width. (b) Comparison of the rim area (obtained by 3D optic disc photography and optical coherence tomography), superior rim width, rim disc area ratio, and average retinal nerve fiber layer (RNFL) thickness. As these parameters were negatively correlated with DDLS, the polarity of the classifiers output was swapped (reversed).

The area under the receiver operating characteristic (ROC) curve was calculated to assess diagnostic probability (Table 4, Figure 2). When the glaucoma group was compared with the combined glaucoma suspect and normal groups, the DDLS of the glaucoma specialist had the best predictive power (0.941), followed by the DDLS obtained by 3D optic disc photography (0.931). The predictive powers of the vertical C/D ratio and C/D area ratio (obtained by 3D optic disc photography) were 0.842 and 0.832, respectively, revealing the overall high predictability of these parameters. The high predictive power of the glaucoma specialist and 3D optic disc photography DDLS evaluations remained when the glaucoma suspect group was removed and the glaucoma and the normal groups were compared and when the combined glaucoma suspect and glaucoma groups were compared with the normal group (Table 4). Rim area measured by 3D optic disc photography and OCT was negatively correlated with DDLS; thus, the area under the curve was calculated from 1 minus the original value, resulting in values of 0.805 and 0.871, respectively. Of the Cirrus OCT parameters, the rim area had the highest predictability (0.871) followed by average RNFL thickness (0.832).

## 4. Discussion

Our findings demonstrate that the interobserver agreement on the DDLS is acceptable among trainees and specialists alike, suggesting that the grading system is a reliable indicator of the morphologic characteristics of the optic nerve head. Furthermore, DDLS measured automatically by 3D optic disc photography was reliable and showed moderate agreement (weighted kappa value, 0.59) with that of the glaucoma specialist. The automatic calculation of DDLS together with various parameters obtained by 3D optic disc photography showed good predictability in the diagnosis of glaucoma. Moreover, we incorporated a supervised teaching module into our training program to increase the interobserver agreement on the DDLS among residents. Our results showed that the training module increased the interobserver agreement kappa statistic, and the effect was greater in the junior (PGY 1 or 2) than in the senior (PGY 3 or 4) residents. We found that agreement among examiners was the poorest on DDLS stages 5 and 6, whereas the concordance was acceptable on stages 0–4 suggesting that caution is required when evaluating the thinnest point of the optic disc rim when using this grading system. Moreover, as the optic disc size must be measured before using the DDLS, care should be taken to measure the optic disc size as accurately as possible.

A previous study found that reliable interobserver agreement on the DDLS was greater than that for C/D ratio on 3D optic disc photographs [10]. However, the sample size in that study was small, and the grading was performed by a glaucoma specialist whose evaluation was likely to have been highly reproducible. Moreover, the previous study used a stand-mounted stereo viewer rather than a 3D display, which is more commonly used at present. Our study was conducted in a clinical setting where the procedure was performed under slit-lamp biomicroscopy to provide more detailed information for the DDLS. Our findings suggest that, unlike C/D ratio, the DDLS grading system takes the thickness of the neural rim and optic disc size into consideration, which may enhance the assessment of the glaucomatous change of the optic nerve head.

C/D ratio assessment using stereographic tests or ophthalmoscopic examination relies heavily on the subjective judgment of the examiner and may limit sensitivity in the detection of microscopic or local changes in the optic disc. Individual variations in the optic disc constitute a further challenge in differentiating between normal and glaucomatous optic discs. Moreover, the conventional ophthalmoscopic method of measuring the C/D ratio cannot differentiate between optic discs with the same C/D ratio but different neural rim thickness and symmetry [6]. Thus, optic disc evaluation using a systematic grading system such as the DDLS increases objectivity and improves diagnostic accuracy for glaucoma.

The 3D retinal camera is a 12-megapixel single-lens reflex camera with three modes: normal, small pupil (non-mydriatic), and stereographic. In the stereographic mode, the picture is taken in the range of 34 degrees, and a 3D image can be captured in a single shot without changing the position of the camera. The 3D images can be visualized on a 2D monitor and both automatic and manual settings of optic nerve and cup boundaries are possible. The stereographic mode has several options including the DDLS. To our knowledge, no previous study has evaluated the performance of the automated DDLS calculation using the camera itself. Our findings indicate that the automated DDLS calculation showed good agreement with the glaucoma specialist and has excellent predictability for the diagnosis of glaucoma. Furthermore, vertical C/D ratio and rim disc area ratio measured by 3D optic disc photography showed good predictability for the diagnosis of glaucoma.

Bayer et al. [11] found a strong correlation between the DDLS and glaucomatous visual field damage. Abdul Majid et al. [12] reported that the DDLS was a useful index for the diagnosis of glaucoma and was highly correlated with indices measured by the visual field test, C/D ratio, and OCT. In a study comparing the optic disc parameters of 3D optic disc photography and Heidelberg retina tomography, Januschowski et al. [13] found that the mean differences were within a tolerable range. Our study extends previous findings by demonstrating that the indices obtained by 3D optic disc photography were significantly correlated with the DDLS evaluation by a glaucoma specialist; thus, providing further support to the usefulness of stereometric parameters in the diagnosis of glaucoma.

Choi and Lee [14] found that 2D optic disc photography relied on the course of the small vessels and shadowing of adjacent structures to measure cup depth and, as such, is prone to subjective findings. However, 3D optic disc photography is relatively free of subjective error because it enables direct visualization of depth. O'Connor et al. [15] reported that the diagnostic accuracy of the quantitative optic disc analysis was considerably higher in 3D optic disc photography than in 2D photography. The software package used in our study automatically calculated the cup length, depth, and ratio, the results of which were more accurate than the bare eye alone. We found that the area under the ROC curve for vertical C/D ratio (0.842), rim disc area ratio (0.833), and C/D area ratio (0.832) obtained by the 3D optic disc photography were high. Moreover, the DDLS calculated by 3D optic disc photography had excellent diagnostic predictability for glaucoma which was almost equal to that of the glaucoma specialist.

A noteworthy finding was that the weighted kappa values of all residents, regardless of PGY, improved after attending a training module. Although there were poor performers in both junior and senior groups, the positive effect of the training program was apparent regardless of level of residency training, suggesting that mandatory attendance in the teaching module would likely improve the reliability and interobserver agreement among ophthalmologists. We found that before attending the teaching module, several of the residents did not measure the size of the optic disc before describing the DDLS. Moreover, when the optic disc size was measured, some residents did not multiply it by the corrective factor, which is necessary when using a 60 or 90 diopter lenses. Thus, the results were inconsistent and inaccurate. However, our findings indicate that these issues can be partially overcome by participation in a structured training program.

In our study, the DDLS was scored by a glaucoma specialist and by residents at different levels of training, which is often the case in busy outpatient clinics faced with limited resources. The value of good interobserver agreement under these challenging conditions cannot be overestimated. Moreover, the evaluation of time-dependent changes in the optic nerve head requires good interobserver agreement on the DDLS. Bearing this in mind, our study showed that a well-structured training program can increase the weighted kappa values for agreement on the DDLS between residents and a glaucoma specialist. We did not initially plan to include a training module in our retrospective study; however, while this study was ongoing, we speculated on the value of a structured training program to improve interobserver agreement on the DDLS among residents. Introduction of such a program was possible because the study was conducted at a tertiary referral center that provides comprehensive training programs for junior doctors.

Our findings highlight the importance of a supervised training program when assessing glaucoma patients using the DDLS and demonstrate the validity of the various parameters measured using 3D optic disc photography for the diagnosis of glaucoma. The various parameters obtained from this camera showed good correlation with those of more commonly used instruments, such as OCT. Future studies are necessary to investigate intraobserver agreement on the DDLS among glaucoma specialists, particularly for cases of stable or progressive glaucoma.

## Figures and Tables

**Table 1:** Patient demographics and baseline characteristics (*n* = 190).

	Normal	Glaucoma suspect	Glaucoma	*P* value^§^
Number (eyes)	40	70	80	
Gender (male/female)	19/21	33/37	40/40	
Visual acuity	0.93 ± 0.14	0.84 ± 0.17	0.64 ± 0.27	<0.001
Refraction error (D)	−0.75 ± 3.03	−0.40 ± 2.40	−0.25 ± 2.56	0.650
Age (year ± SD)	38.97 ± 11.60	49.48 ± 13.19	60.89 ± 14.69	<0.001
DDLS* (glaucoma specialist)	1.47 ± 0.56	2.39 ± 0.78	4.91 ± 1.42	<0.001
DDLS* (3D Optic disc photography)	0.58 ± 0.50	2.03 ± 0.66	4.23 ± 1.23	<0.001
MD^†^ (dB ± SD)	−2.35 ± 3.28	−2.47 ± 2.52	−8.57 ± 8.78	<0.001
CSP^‡^ (dB ± SD)	1.60 ± 0.40	1.69 ± 1.17	4.18 ± 3.38	<0.001
Disc diameter (mm)	1.41 ± 0.15	1.48 ± 0.18	1.44 ± 0.16	0.451

*Disk damage likelihood scale.

^†^Mean deviation in visual field.

^‡^Corrected pattern standard deviation.

^§^Statistical significance, ANOVA.

**Table 2:** Mean values derived from the optic nerve head ophthalmoscopic examination, disc damage likelihood scale, and optical coherence tomography according to diagnosis.

	Normal	Glaucoma suspect	Glaucoma	*P* value*	Post hoc analysis		
					Normal versus Glaucoma suspect	Normal versus Glaucoma	Glaucoma suspect versus Glaucoma
*DDLS evaluated using 3D optic disc photography *	0.58 ± 0.50	2.03 ± 0.66	4.23 ± 1.23	<0.001	<0.01	<0.001	<0.001
Ophthalmoscopic examination.							
Horizontal C/D ratio	0.37 ± 0.10	0.61 ± 0.11	0.63 ± 0.18	<0.001	<0.001	<0.001	0.673
Vertical C/D ratio	0.38 ± 0.09	0.57 ± 0.13	0.59 ± 0.20	<0.001	<0.001	<0.001	0.590
3D optic disc photography							
Vertical C/D ratio	0.48 ± 0.11	0.57 ± 0.03	0.64 ± 0.11	<0.001	<0.001	<0.001	<0.001
Superior rim width	0.45 ± 0.10	0.41 ± 0.08	0.32 ± 0.09	<0.001	0.182	<0.001	<0.001
Inferior rim width	0.47 ± 0.12	0.44 ± 0.10	0.36 ± 0.13	<0.001	0.361	<0.001	0.001
Cup area	0.68 ± 0.35	1.04 ± 0.20	1.38 ± 0.74	<0.001	<0.001	<0.001	0.005
Disc area	2.55 ± 0.55	3.17 ± 0.24	3.10 ± 0.89	<0.001	<0.001	0.002	0.657
Rim area	1.87 ± 0.35	2.09 ± 0.20	1.71 ± 0.41	<0.001	0.014	0.089	<0.001
C/D area ratio	0.25 ± 0.10	0.33 ± 0.65	0.42 ± 0.12	<0.001	0.002	<0.001	<0.001
Rim disc area ratio	0.74 ± 0.10	0.66 ± 0.06	0.57 ± 0.12	<0.001	0.002	<0.001	<0.001
Cup volume	0.11 ± 0.07	0.31 ± 0.17	0.37 ± 0.34	<0.001	<0.001	<0.001	0.285
Disc volume	0.76 ± 0.33	1.49 ± 0.32	1.39 ± 1.01	<0.001	<0.001	0.005	0.352
Rim volume	0.35 ± 0.17	0.64 ± 0.33	0.32 ± 0.23	<0.001	<0.001	0.669	0.001
Mean cup depth	0.18 ± 0.13	0.31 ± 0.15	0.36 ± 0.34	<0.001	0.001	0.005	0.486
Maximum cup depth	0.51 ± 0.29	1.43 ± 1.61	1.10 ± 1.39	0.019	0.010	0.018	0.326
Height variation contour	0.81 ± 0.71	1.25 ± 1.34	1.25 ± 1.35	0.234	0.281	0.101	0.994
Cirrus OCT RNFL and optic nerve head analysis							
Rim area	1.20 ± 0.28	1.14 ± 0.22	0.868 ± 0.33	<0.001	0.136	<0.001	<0.001
Disc area	1.92 ± 0.40	2.51 ± 0.42	2.26 ± 0.56	<0.001	<0.001	0.008	0.009
Average C/D ratio	0.54 ± 0.14	0.71 ± 0.06	0.74 ± 0.14	<0.001	<0.001	<0.001	0.277
Vertical C/D ratio	0.50 ± 0.13	0.68 ± 0.05	0.72 ± 0.14	<0.001	<0.001	<0.001	0.050
Cup volume	0.20 ± 0.12	0.53 ± 0.23	0.55 ± 0.34	<0.001	<0.001	<0.001	0.646
Average RNFL thickness	95.33 ± 8.61	91.60 ± 9.56	76.48 ± 16.30	<0.001	0.167	<0.001	<0.001

*Statistical significance was tested by ANOVA.

OCT: optical coherence tomography.

C/D ratio: cup/disc ratio.

RNFL: retinal nerve fiber layer.

**Table 3:** Correlations between the DDLS and stereometric parameters obtained using 3D optic disc photography.

	r*	*P*
C/D ratio		
Vertical	0.733	<0.001
Horizontal	0.794	<0.001
Visual field		
MD	−0.561	<0.001
CPSD	0.498	<0.001
Parameters obtained using 3D optic disc photography		
Vertical C/D ratio	0.623	<0.001
Superior rim width	−0.548	<0.001
Inferior rim width	−0.433	<0.001
Cup area	0.526	<0.001
Disc area	0.107	0.289
Rim area	−0.419	<0.001
C/D area ratio	0.618	<0.001
Rim disc area ratio	−0.618	<0.001
Cup volume	0.384	<0.001
Disc volume	0.049	0.629
Rim volume	−0.410	<0.001
Mean cup depth	0.041	0.682
Max cup depth	0.075	0.458
Height variation	−0.171	0.089
Parameters acquired using OCT RNFL and optic nerve head analyses		
Rim area	−0.748	<0.001
Disc area	0.087	0.389
Average C/D ratio	0.706	<0.001
Vertical C/D ratio	0.739	<0.001
Cup volume	0.541	<0.001
Average RNFL thickness	−0.701	<0.001

MD: mean deviation.

CPSD: corrected pattern standard deviation.

C/D ratio: cup/disc ratio.

OCT: optical coherence tomography.

*Correlations were evaluated using Pearson's correlation coefficient.

**Table 4:** Calculation of the area under the curve for each parameter according to group constellation.

Test result variable(s)	Glaucoma versus glaucoma suspect and normal	Glaucoma versus normal (glaucoma suspect removed)	Glaucoma and glaucoma suspect versus normal
DDLS obtained by 3D optic disc photography	0.931	0.974	0.915
DDLS scored by the glaucoma specialist	0.941	0.992	0.961
Vertical C/D ratio	0.842	0.896	0.873
*Superior rim width	0.828	0.852	0.751
*Inferior rim width	0.762	0.800	0.725
Cup area	0.746	0.868	0.856
Disc area	0.445	0.668	0.738
*Rim area	0.805	0.715	0.546
C/D area ratio	0.832	0.897	0.850
*Rim disc area ratio	0.833	0.898	0.851
Cup volume	0.691	0.893	0.887
*Rim volume	0.758	0.593	0.591
Rim area (OCT)	0.871	0.914	0.819
Cup volume (OCT)	0.680	0.879	0.876
Average RNFL (OCT)	0.832	0.874	0.780

DDLS: disc damage likelihood scale.

C/D: cup/disc.

OCT: optical coherence tomography.

*Parameters were negatively correlated with DDLS; thus, the area under the curve was obtained from 1 minus the original value.

## References

[1] Foster PJ, Buhrmann R, Quigley HA, Johnson GJ (2002). The definition and classification of glaucoma in prevalence surveys. *British Journal of Ophthalmology*.

[2] Quigley HA, Addicks EM, Green WR (1982). Optic nerve damage in human glaucoma. III. Quantitative correlation of nerve fiber loss and visual field defect in glaucoma, ischemic neuropathy, papilledema, and toxic neuropathy. *Archives of Ophthalmology*.

[3] Quigley HA, Dunkelberger GR, Green WR (1989). Retinal ganglion cell atrophy correlated with automated perimetry in human eyes with glaucoma. *American Journal of Ophthalmology*.

[4] Sommer A, Katz J, Quigley HA (1991). Clinically detectable nerve fiber atrophy precedes the onset of glaucomatous field loss. *Archives of Ophthalmology*.

[5] Spaeth GL, Henderer J, Liu C (2002). The disc damage likelihood scale: reproducibility of a new method of estimating the amount of optic nerve damage caused by glaucoma. *Transactions of the American Ophthalmological Society*.

[6] Henderer JD (2006). Disc damage likelihood scale. *British Journal of Ophthalmology*.

[7] Danesh-Meyer HV, Gaskin BJ, Jayusundera T, Donaldson M, Gamble GD (2006). Comparison of disc damage likelihood scale, cup to disc ratio, and Heidelberg retina tomograph in the diagnosis of glaucoma. *British Journal of Ophthalmology*.

[8] Hornová J, Navarro JBVK, Prasad A, Freitas DGJ, Nunes CM (2008). Correlation of disc damage likelihood scale, visual field, and Heidelberg Retina Tomograph II in patients with glaucoma. *European Journal of Ophthalmology*.

[9] Fleiss JL (1981). *Statistical Methods for Rates and Proportions*.

[10] Henderer JD, Liu C, Kesen M (2003). Reliability of the disk damage likelihood scale. *American Journal of Ophthalmology*.

[11] Bayer A, Harasymowycz P, Henderer JD, Steinmann WG, Spaeth GL (2002). Validity of a new disk grading scale for estimating glaucomatous damage: correlation with visual field damage. *American Journal of Ophthalmology*.

[12] Abdul Majid ASB, Kwag JH, Jung SH, Yim HB, Kim YD, Kang KD (2010). Correlation between disc damage likelihood scale and optical coherence tomography in the diagnosis of glaucoma. *Ophthalmologica*.

[13] Januschowski K, Blumenstock G, Rayford CE, Bartz-Schmidt K-U, Schiefer U, Ziemssen F (2011). Stereometric parameters of the optic disc. Comparison between a simultaneous non-mydriatic stereoscopic fundus camera (KOWA WX 3D) and the Heidelberg scanning laser ophthalmoscope (HRT IIII). *Ophthalmologe*.

[14] Choi YR, Lee SH (1999). Optic disc measurements with stereophotograph in normal eyes. *Journal of the Korean Ophthalmological Society*.

[15] O’Connor DJ, Zeyen T, Caprioli J (1993). Comparisons of methods to detect glaucomatous optic nerve damage. *Ophthalmology*.

